# Urine and serum metabolomic profiling reveals that bile acids and carnitine may be potential biomarkers of primary biliary cirrhosis

**DOI:** 10.3892/ijmm.2015.2233

**Published:** 2015-06-03

**Authors:** YING-MEI TANG, JIA-PING WANG, WEI-MIN BAO, JIN-HUI YANG, LIN-KUN MA, JING YANG, HUI CHEN, YING XU, LI-HONG YANG, WEN LI, YAN-PING ZHU, JI-BIN CHENG

**Affiliations:** 1 Department of Gastroenterology, The Second Affiliated Hospital of Kunming Medical University, Yunnan Research Center for Liver Diseases, Kunming, Yunnan, P.R. China; 2 The Second Affiliated Hospital of Kunming Medical University, Kunming, Yunnan, P.R. China; 3 Department of General Surgery, Yunnan Provincial First People’s Hospital, Kunming, Yunnan, P.R. China

**Keywords:** metabolomic profiling, primary biliary cirrhosis, biomarkers, bile acids, carnitine

## Abstract

In order to provide non-invasive, reliable and sensitive laboratory parameters for the diagnosis of primary biliary cirrhosis (PBC), metabolic technology of ultraperformance liquid chromatography coupled with quadrupole-time-of-flight mass spectrometry (UPLC/Q-TOF MS) was used to compare small molecule metabolites in blood and urine from patients with PBC and healthy controls. We then screened for biomarkers in the blood and urine of the patients with PBC. Data were processed by Bruker ProfileAnalysis metabonomic software and imported to SIMCA-P software, which utilized principal component analysis (PCA) to create models of patients with PBC and healthy controls. In total, 18 urinary markers were found and the levels of 11 of these urinary markers were elevated in the patients with PBC, whereas the levels of the remaining 7 markers were lower in the PBC group compared to the control group. We also identified 20 blood-based biomarkers in the patients with PBC and the levels of 9 of these markers were higher in the PBC group, whereas the levels of the remaining 11 markers were lower in the patients with PBC compared to the controls. Among these biomarkers, the levels of bile acids increased with the progression of PBC, while the levels of carnitines, such as propionyl carnitine and butyryl carnitine, decreased with the progression of PBC. In conclusion, the findings of the present study suggest that the circulating levels of bile acids and carnitine are differentially altered in patients with PBC.

## Introduction

Primary biliary cirrhosis (PBC) is a chronic liver-specific autoimmune disorder with an unidentified etiology. It mainly affects middle-aged women (male-to-female rario, 1:9) and is categorized by the infiltration of lymphocytes in portal tracts, the destruction of small- and medium-sized intrahepatic bile ducts, and the progressive scarring that initially leads to fibrosis and, eventually, to cirrhosis and end-stage hepatic failure over a period of 10–20 years without treatment (1,2). Previous data have indicated that PBC, particularly asymptomatic PBC, is no longer considered a rare disease due to diagnostic improvements that include biochemical tests, histological analyses and the detection of autoantibodies in serum (3,4). Although histopathological changes serve as the ‘gold standard’ for the diagnosis of PBC, a liver biopsy is an invasive, painful and costly procedure that is associated with the possibility of sampling error and variability in interpretation. To date, biomarkers for the diagnosis of PBC, such as anti-mitochondrial antibody (AMA) have been evaluated. AMA, which reacts with the pyruvate dehydrogenase E2 subunit, is commonly accepted as a serological hallmark for the diagnosis of PBC, since AMA appears in approximately 90% of patients with PBC (5). However, depending on the assay used, up to 15% of patients with PBC have been found to be AMA-negative (5). Furthermore, although some AMA-negative patients are positive for antinuclear antibody (ANA) components in PBC, unlike AMA, which is used for diagnosis, PBC-associated ANA correlates with the disease severity and may thus serve as a marker for poor prognosis instead of diagnosis (5). Besides, although the nuclear components, including Sp100, promyelocytic leukemia proteins and two components of the nuclear pore complex protein (gp210 and nucleoporin 62), react with ANA, as has been previously demonstrated, anti-sp100 antibody is not a better prognostic marker for Chinese patients with PBC compared to anti-gp210 antibody, which was only detected in 34.3% of Chinese patients with PBC (6).

Therefore, it is still necessary to discover other novel biomarkers for PBC. Metabolomics is the study of a large number of low molecular weight metabolites, including amino acids, hormones and sugars, and has arisen as a potent tool for discovering novel biomarkers for Parkinson’s disease (7), prostate cancer (8), type 2 diabetes (9), acute myocardial infarction (10) and preeclampsia (11). Metabolomics has also provided some important insight into the pathogenesis of human non-alcoholic fatty liver disease, non-alcoholic steatohepatitis and PBC (12–16). In this study, we utilized a metabonomics technique based on ultraperformance liquid chromatography coupled with quadrupole-time-of-flight mass spectrometry (UPLC/Q-TOF MS) in an aim to discover novel markers for PBC and elucidate their pathological roles in the progression of PBC.

## Subjects and methods

### Patients

A total of 32 patients with a clinical and/or histological diagnosis of PBC at the Second Affiliated Hospital of Kunming Medical University, Kunming, China between May 2010 and November 2011 were enrolled in this study. The experimental protocol was established, according to the ethical guidelines of the Helsinki Declaration and was approved by the Human Ethics Committee of the Affiliated Hospital of Kunming Medical University. Written informed consent was obtained from each participant prior to enrollment. The diagnosis of PBC was based on the following criteria, as previously described (17): i) increased levels of biochemical markers reflecting intrahepatic cholestasis for >6 months; ii) an normal biliary system, as shown by ultrasound or cholangiography; iii) patients were serum AMA- or AMA-M2 positive; iv) patients were serum AMA/AMA-M2-negative, but a liver biopsy revealed moderate or severe periportal or periseptal inflammation. The appropriate exclusion criteria included other liver diseases, such as alcoholic liver disease, viral hepatitis, drug-induced liver diseases, genetic diseases, cancer, pregnancy, lactating subjects, as well as subjects whose blood or urine specimen samples were kept at room temperature for >30 min. All 32 healthy control subjects (HCs) were confirmed to have normal liver function and fit the same exclusion criteria as the patients with PBC. Informed consent was obtained from all subjects. The study protocol conformed to the ethical guidelines of the 2008 Declaration of Helsinki and was approved by the research ethics committee of our institution.

### Sample collection and metabolomics analysis

In all subjects, blood (3 ml) was drawn after a 12-h fast, serum was obtained by centrifugation of the blood samples at 3,000 rpm at 4°C for 10 min, and urina sanguinis samples (2 ml) were collected on the same day as the blood samples. All the samples were stored at −80°C until subsequent analyses. The urine samples were thawed at room temperature and centrifuged at 10,000 × g at 4°C for 20 min. Subsequently, 150 *µ*l of the supernatant were diluted with purified water to 1 ml and filtered through a 0.22-*µ*l filter. A total of 10 *µ*l of sample was then taken. The blood samples were thawed at room temperature, and 180 µl of the sample were then added to acetonitrile (720 *µ*l) followed by vigorous shaking for 30 sec and centrifugation at 15,000 × g at 4°C for 10 min. The supernatant was stored at 4°C for analysis within 48 h.

### Chromatography

Chromatographic separation was performed on a 15 cmx2.1 mm Acquity™ 1.8 *µ*m C18 column (Agilent Technologies, Santa Clara, CA, USA) using an Acquity™ ultra performance liquid chromatography system (Ultimate 3000-Bruker mXis; Dionex, Sunnyvale, CA, USA). A 10-*µ*l aliquot of each sample was injected into the column. The column was maintained at 4°C and eluted with 0.1% formic acid (A) and acetonitrile (B) in a linear gradient (0–60 min, 5–100% of B). The follow rate was at 0.3 ml/min.

### Mass spectrometry

Mass spectrometry was performed on a Waters Q-TOF micro Mass Spectrometer (Waters MS Technologies, Manchester, UK) in both the ESI^+^ and ESI^−^ ion modes. The following parameters were used: nebulization gas, 6 l/min at 200°C; cone, 50 l/h; source gas temperature, 100°C; capillary voltage, 4,500 V; cone voltage, 35 V; Q-TOF micro MS acquisition rate, 0.5 sec with a 0.1 sec interscan delay. The scan range was from 50 to 1,000 m/z. Data were collected in centroid mode. All analyses were acquired using the lock spray to ensure accuracy and reproducibility; sodium formate was used as the lock mass at a concentration of 1 mmol/l and a flow rate of 10 *µ*l/min, deriving an [M + H]^+^ ion at 4,500 V in ESI^+^ mode, and an [M - H]^−^ ion at 3,200 V in ESI^−^ mode. The lock spray frequency was set at 20 sec. Sodium formate was used as an internal control in this assay.

### Data analysis

The raw data were analyzed using ProfileAnalysis software (Ultimate 3000-Bruker mXis; Dionex) the retention time (t_r_) and m/z data pair for each peak were detected using software from Umetrics AB (Umeå, Sweden). The ion intensities for each peak detected were then normalized within each sample to the sum of the peak intensities in that sample, as previously described (18). The resulting normalized peak intensities were then multiplied by 10,000. The data were then exported and analyzed by principal components analysis (PCA) and partial least squares-discriminate analysis (PLS-DA) using SIMCA-P software (Umetrics AB). The statistical analysis was performed using SPSS version 10.0 software (SPSS, Inc., Chicago, IL, USA). A P-value <0.05 was considered to indicate a statistically significant difference.

## Results

### Characteristics of the patients

**Figure 1:**
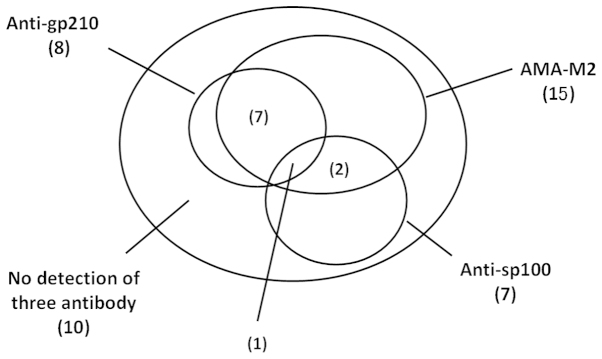
Detection of autoantibodies in serum from patients with primary biliary cirrhosis (PBC). A proportional Venn diagram for the patients with PBC enrolled in present study is presented, showing the frequency of AMA-M2, Anti-gp210 and Anti-sp100 antibody detection.

The characteristics of 32 PBC patients and the 32 HCs were analyzed. Due to hemolysis, one blood sample from each group was excluded for further assessment. The mean ages of the patients with PBC and the HCs were 52.6±11.95 years (range, 30–76 years) and 52.1±14.6 years (range, 27–76 years), and the female-male ratios were 27/5 and 25/7, respectively. There were no significant differences between the patients with PBC and the HCs in terms of age, parity and gender (P>0.05). Sixteen cases from the PBC group were newly diagnosed with PBC, and the mean time course of the disease was 25.4±45.3 months (range, 4 days to 17 years). The liver function Child-Pugh score was grade A in 23 cases, grade B in 8 cases and grade C in 1 case. The globulin and transaminase levels are presented Table I. The detection levels of autoantibodies in the sera from patients with PBC are shown in Fig. 1. In total, 15 patients were AMA-M2-positive, 8 were anti-gp210-positive, 7 were anti-sp100-positive, 10 were AMA-M2-, anti-gp210- and anti-sp100-negative, 1 was AMA-M2-, anti-gp210- and anti-sp100-positive, 7 were AMA-M2- and anti-gp210-positive, 1 was anti-gp210- and anti-sp100-positive, and 2 were AMA-M2- and anti-sp100-positive.

### Urine and serum profiles in patients with PBC

**Figure 2:**
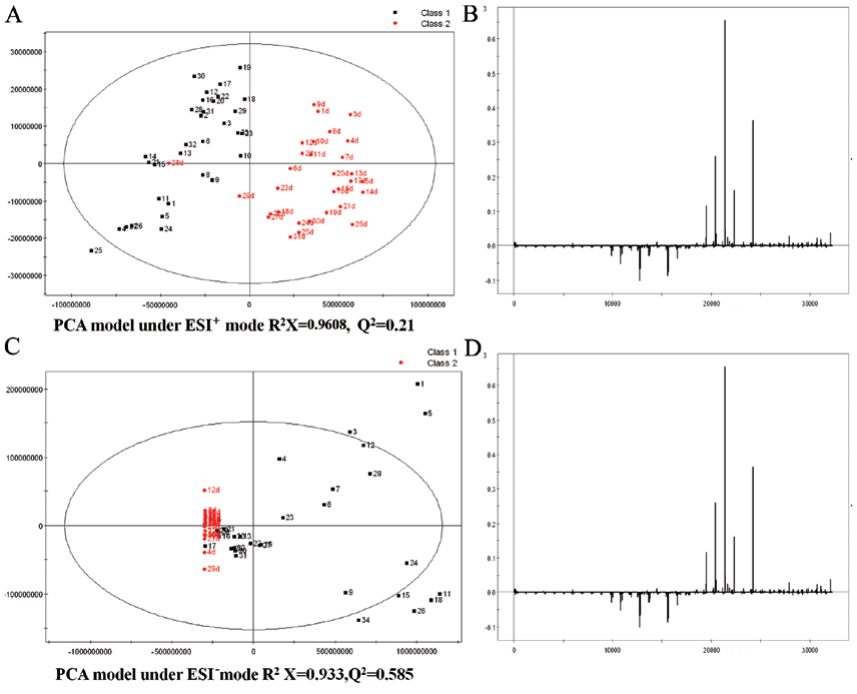
Principal component analysis (PCA) score plot (A and C) and loading plots (B and D) of the urine metabolome from patients with primary biliary cirrhosis (PBC) and healthy control subjects (HCs) scanned by ESI^+^ and ESI^−^, respectively. Class 1, PBC group; class 2, HC group.

**Figure 3:**
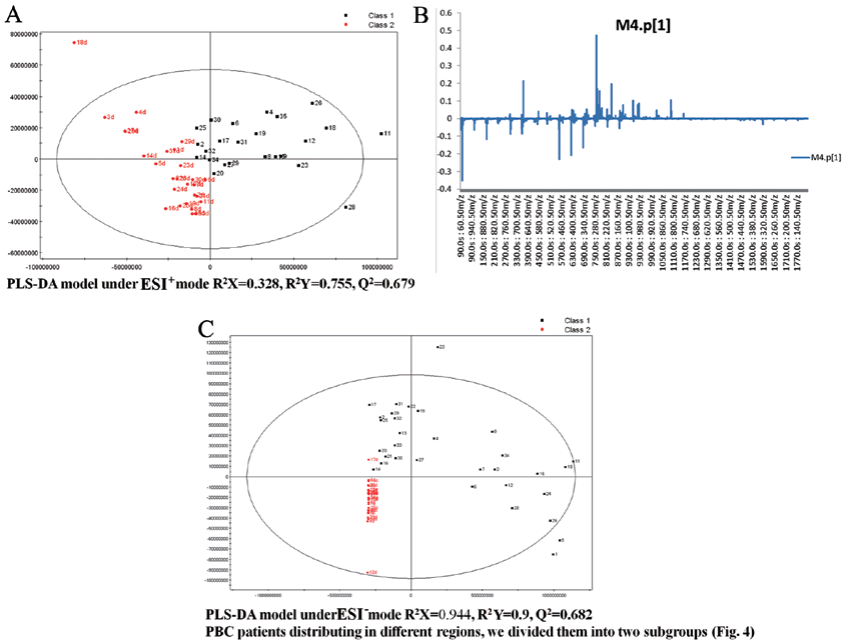
Partial least squares discriminate analysis (PLS-DA) score plot (A and C) and loading plots (B) of the urine metabolome from patients with primary biliary cirrhosis (PBC) and healthy control subjects (HCs) scanned by ESI^+^ and ESI^−^, respectively. Patients with PBC were divided into 2 subgroups, since they were distributed in different regions (see Fig. 4). Class 1, PBC group; class 2, HC group.

**Figure 4:**
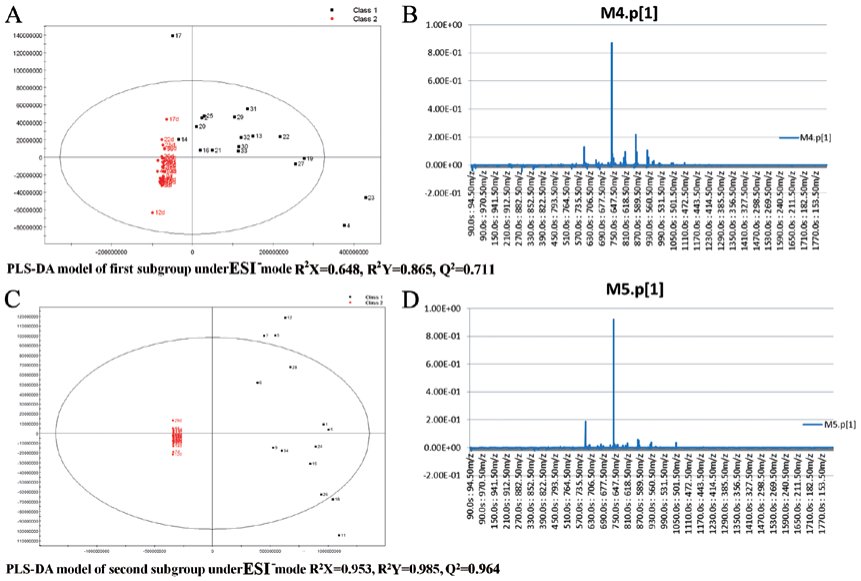
Partial least squares discriminate analysis (PLS-DA) score plot (A and C) and loading plots (B and D) of the urine metabolome from patients with primary biliary cirrhosis (PBC; 2 subgroups) and healthy control subjects (HCs) scanned by ESI^−^. Class 1, PBC group; class 2, HC group.

Following UPLC/Q-TOF analysis, the retention time and m/z data pair for each peak were detected. Although PCA is an excellent tool for data reduction and hence graphical display, it does not lend itself to the development of a diagnostic model to predict the presence or absence of disease. To address this issue, PLS-DA and the coefficient of correlation analysis were used for marker selection and identification, as previously described (19). Figs. 2–4 show the score plots of PCA and PLS-DA of the urine metabolome from the patients with PBC and the HCs scanned by ESI^+^ and ESI^−^. The prediction rate and resolution are very good under the urine ESI mode to distinguish the PBC group from the control group using the PLS-DA model. Since patients with PBC were shown to be distributed in different regions, we divided them into 2 subgroups.

**Figure 5:**
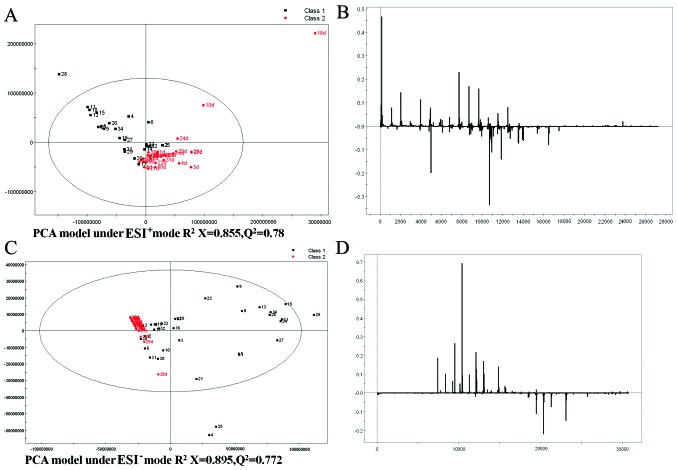
Principal component analysis (PCA) score plot (A and C) and loading plots (B and D) of the serum metabolome from patients with primary biliary cirrhosis (PBC) and healthy control subjects (HCs) scanned by ESI^−^ and ESI^+^, respectively. Class 1, PBC group; class 2, HC group.

**Figure 6:**
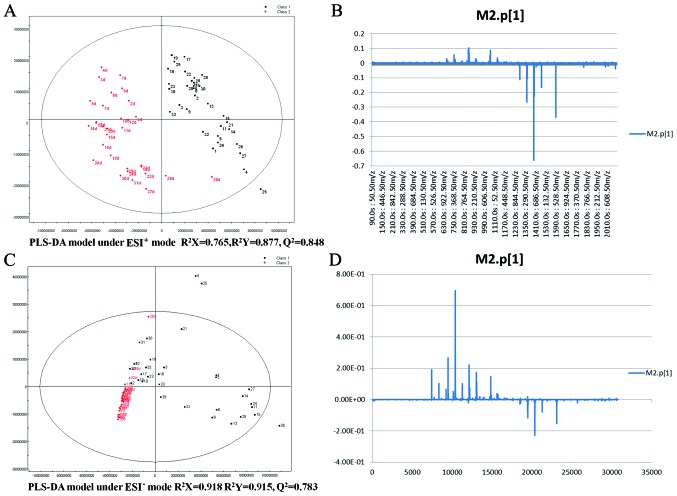
Partial least squares discriminate analysis (PLS-DA) score plot (A and C) and loading plots (B and D) of the serum metabonome from patients with primary biliary cirrhosis (PBC) and healthy control subjects (HCs) scanned by ESI^−^ and ESI^+^, respectively. Class 1, PBC group; class 2, HC group.

Figs. 5 and 6 show the score plots of PCA and PLS-DA of the serum metabolome from patients with PBC and the HCs scanned by ESI^+^ and ESI^−^. In both ion scanning modes, the modeling parameters listed in Table II indicate that the models of PBC had been successfully established and the patients with PBC could be easily distinguished through these models. Potential biomarkers were identified in the urine (Table III) and serum (Table IV) from PBC patients. The levels of 11 of the 18 potential biomarkers identified were increased in the urine of the patients with PBC, while the levels of 7 of these 18 potential biomarkers were decreased in the urine of the patients with PBC compared to the HCs. Similarly, the levels of 9 of the 20 potential biomarkers identified in the serum of the patients with PBC were increased, while the levels of 11 of these 20 potential biomarkers were decreased in the serum of the patients with PBC compared to the HCs. Among these biomarkers, the levels of bile acids increased with the progression of PBC, while the levels of carnitines, such as propionyl carnitine and butyryl carnitine, decreased with the progression of PBC.

Small molecule metabolites may be distinguished effectively using PCA and PLS-DA in the ESI^+^ and ESI^−^ mode. This method is a good prediction method, apart from PCA-X of the urine ESI^+^ mode (R^2^X=0.608, Q^2^=0.21). All the identified metabolites of the potential biomarkers are listed in Tables III and IV.

## Discussion

Significant hurdles to the early diagnosis of PBC still exist. Due to the shortcomings of liver biopsies and the limitations of AMA and ANA antibody assays, a cost-effective early detection method will require a very accurate screening method. Therefore, clinically helpful biomarkers for the early detection of PBC should be measurable in an easily accessible bodily fluid, such as blood or urine. Furthermore, these biomarkers should provide predictive value with high specificity and sensitivity. In the present study, we compared the metabolomics profiles of urine and serum from patients with PBC with those of age- and gender-matched HCs using both unsupervised PCA and supervised PLS-DA with UPLC/Q-TOF.

In this study, the analysis of the urine and serum metabolomics profiles identified important differences between the patients with PBC and the HCs. One of the most significant observations was that the levels of bile acids were significantly increased in the patients with PBC compared to the HCs. In line with our results, in a previous study, the concentrations of total bile acids, taurine and glycine conjugates of primary bile acids were increased in patients with PBC, compared to non-cholestatic donors (16). Indeed, bile acids are the main product of endogenous cholesterol metabolism and are related to lipid absorption and biliary excretion. Bile acids endure a strong enterohepatic recirculation, through which they can be converted into secondary deoxycholic and lithocholic acids in the intestines. Bile acids reabsorbed in the intestines may be further absorbed back into the liver, as only a small fraction of bile acids is found in the peripheral circulating blood and urine in healthy individuals. However, liver injury caused by liver diseases, such as cirrhosis and gallbladder disease, results in a decrease in the hepatic clearance of bile acids and, eventually, in an increase in the levels of bile acids in the serum. Therefore, bile acids are considered to be a hallmark of liver injury. During the early stages of liver cirrhosis, bile acids may induce the upregulation of hepatocyte-derived monocyte chemotaxis protein-1 (MCP-1), a hepatic stellate cell-responsive chemokine, leading to hepatic stellate cell recruitment. In turn, MCP-1 mediates hepatic stellate cell recruitment, causing a further decrease in the hepatic clearance of bile acids and further promoting liver cirrhosis (20). Furthermore, bile acids directly activate the proinflammatory signaling network in hepatocytes and induce the upregulation of multiple proinflammatory mediators, such as cytokines, chemokines, adhesion molecules and other proteins that influence immune cell functions (21). Moreover, bile acid receptor GP-BAR1 (TGR5) expression has been shown to be increased in rodent models of colitis and Crohn’s disease (22); and the treatment of a murine model of non-alcoholic fatty liver disease with a dual bile acid FXR/TGR5 receptor agonist was shown to decrease intrahepatic inflammation and altered the immune phenotype of monocytes (23).

The bile acid sensor farnesoid X receptor (FXR) is required for the immunoregulatory activities of Toll-like receptor-9 in intestinal inflammation (24). Another intriguing difference between patients with PBC and HCs is the relatively higher level of carnitines, such as propionyl carnitine and butyryl carnitine in HCs. Carnitine is a substance necessary for long-chain fatty acids to pass the mitochondrial intramembrane to induce β-oxidation (25). However, the results of studies assessing carnitine metabolism in patients with PBC have been controversial thus far. Some studies have demonstrated that the lack of carnitine decreases the rate of hepatic fatty acid oxidation and may be associated with hepatic steatosis, which causes pathological consequences in the liver (26), whereas elevated carnitine concentrations are present in patients with cirrhosis of different causes (27). In addition, an increased urinary excretion of total carnitine has been found due to an increase in the fractional excretion of both free carnitine and short-chain acylcarnitine (28). Furthermore, severe carnitine deficiency leads to the increased apoptosis of enterocytes, villous atrophy, inflammation and gut injury (29); and L-carnitine supplementation has been shown to have a positive effect in improving immune responses in aged animals (30).

The third potential biomarker for PBC is the relatively higher level of prostaglandin (PG), which is an important player in inflammatory processes. In fact, phytohemagglutinin (PHA)-stimulated-enriched monocytes of patients with PBC produce approximately 3-fold more PGE_2_ than that of normal control monocytes (31); and the PGE_2_ produced in monocytes may play a primary role in the hyporesponsiveness to PHA observed in patients with PBC (32). Moreover, epithelial cells from patients with PBC have been shown to have moderate levels of cyclooxygenase-2 expression, which in turn participates in the conversion of arachidonic acid into PG (33). PGs also function in the transition and maintenance of chronic inflammation. One role that PGs play in such processes is the amplification of cytokine signaling, which in turn facilitates acquired immunity and induces long-lasting immune inflammation (34). PGs also play a part in chronic inflammation by generating a positive feedback loop and/or inducing chemokines and recruiting inflammatory cells to alternate active cell populations in affected tissues (35). In addition, PGs contribute to tissue remodeling in fibrosis in a transforming growth factor β-independent manner (36). Thus, PGs are commonly known as mediators of inflammation (37–39).

The fourth difference between the patients with PBC and the HCs was the elevated level of urine deoxyguanosine, which is known as an oxidatively damaged nucleobase of DNA excreted into the urine. Indeed, the possible association of oxidative stress has been suggested to be involved in the pathogenesis of cellular senescence in PBC (40). For example, oxidative stress and proinflammatory cytokines, such as interferon-β and tumor necrosis factor-α, induce reactive oxygen species generation and activate the ATM/p53/p21WAF1/Cip1 pathway, followed by biliary epithelial senescence in the case of PBC (41).

In conclusion, the findings of the present study suggest that the circulating levels of bile acids and carnitine are differentially altered in patients with PBC. However, due to the limitations of the present pilot study, such as a small patient population and the absence of a stratification metabolomics profile amongst the disease stages of PBC, further studies to confirm the differential variations are warranted.

## Figures and Tables

**Table I:** Clinical data of globulin and transaminase levels in the patients with PBC.

Liver function	No.	Min. value	Max. value	Mean ± SD
ALT (U/l)	32	15	1176	156±225.4
AST (U/l)	32	30	710	145.2±148.46
ALP (U/l)	32	61	893	299±203.2
GGT (U/l)	32	29	1359	346.8±280.67
GLO (g/l)	32	23.6	53.5	34.9±6.56

PBC, primary biliary cirrhosis; ALT, alanine aminotransferase; AST, aspartate transaminase; ALP, alkaline phosphatase; GGT, glutamyltransferase; GLO, globulin; Min., minimum; Max., maximum.

**Table II:** Summary of the modeling information for PCA and PLS-DA analysis.

Sample mode	Analysis mode	R^2^X	R^2^Y	Q^2^
Urine ESI^+^	PCA-X	0.608		0.21
Urine ESI^+^	PLS-DA	0.328	0.755	0.679
Urine ESI^−^	PCA-X	0.933		0.585
Urine ESI^−^a	PLS-DA	0.944	0.9	0.682
Urine ESI-subgroup 1	PLS-DA	0.648	0.865	0.711
Urine ESI-subgroup 2	PLS-DA	0.953	0.985	0.964
Serum ESI^+^	PCA-X	0.855		0.78
Serum ESI^+^	PLS-DA	0.765	0.877	0.848
Serum ESI^−^	PCA-X	0.895		0.772
Serum ESI^−^	PLS-DA	0.918	0.915	0.783

a Patients with PBC were divided into 2 subgroups, since they distributed in different regions. PBC, primary biliary cirrhosis; PCA, principal component analysis; PLS-DA, partial least squares-discriminate analysis.

**Table III:** Selected markers indicating a difference between ESI^+^ and ESI^−^ scans of the urine samples from patients with PBC and HCs.

No	Tr±30 (sec)	Patients with PBC vs. HCs	m/z	Potential markers
ESI^+^				
1	390	↑	321.1362	Three oxygen radicals on cinnamic acid
2	750	↑	414.2997	Glycolic amide
3	810	↑	626.3537	aSugar goes to oxygen cholic acid 3-glucoside, bile acid, glucuronic acid
4	930	↑	432.3118	7a-hydroxy-3-ursodeoxycholic acid oxo-5b
5	930	↑	823.4127	UK
6	1110	↑	593.3328	Urobilinogen
7	90	↓	204.9095	Potassium chlorate, cyano sulfate anion
8	150	↓	218.1390	Propionyl carnitine
9	210	↓	232.1548	Butyryl L-carnitine, butyryl carnitine
10	330	↓	268.1060	Deoxyguanosine, adenosine
11	390	↓	189.0657	Hippuric acid, 3-succinyl pyridine, adrenaline, hydroxybenzoic acid, hydroxybenzoic acid
12	570	↓	286.2012	UK
13	630/690	↓	310.2013	L-group of ammonia and alcohol, L-carnitine zinn
ESI^−^				
14	630	↑	288.6198	aCow bile acid sodium sulfonated goes to oxygen-7-sulfuric acid
15	810	↑	624.3392	aGlycine goes to deoxycholic acid 3-glucoside acid
16	870	↑	471.2423	aUrsodesoxycholic acid, chenodeoxycholic acid 3-sulfuric acid, bile acid sulfate goes to oxygen
17	930	↑	448.3072	aAmmonia bearing bile acid goes to oxygen, chenodeoxycholic acid glycine conjugated, DNA single glycine conjugate
18	750	↑	528.2645	aGlycine goes to deoxycholic acid-3-sulfuric acid, aGlycine lithocholic acid 3-sulfuric acid disodium salt

PBC, primary biliary cirrhosis; HCs, healthy control subjects.

a Denotes bile acids. Unmarked markers represent carnitines. UK, unknown.

**Table IV:** Selected markers indicating a difference between the ESI^+^ and ESI^−^ scans of the serum samples from patients with PBC and the HCs.

No.	Tr±30 (sec)	Patients with PBC vs. HCs	m/z	Potential markers
ESI^+^				
1	670/750	↑	414.3005	UK
2	870/930/1110/1050	↑	450.3213	aCDCA, glycine conjugated, deoxycholic acid f glycine conjugated
3	1050	↑	466.316	aGCA
4	1290/1350	↓	520.394	Salbutamol
5	1350/1410	↓	496.3394	3-β,α-hydroxy-5–7 bile acid ethyl ester, carnitine 3.24 X-hydroxy-3-alcohol
6	1470	↓	522.3556	UK
7	1590	↓	524.2707	UK
8	2070	↓	758.5705	UK
ESI^−^				
9	570/630/690	↑	288.62	aCattle sulfonated goes to oxygen cholic acid-7-sulfuric acid
10	690/750	↑	528.2632	aGlycine goes to deoxych olic acid-3-sulfuric acid, aGlycine lithocholic acid 3-sulfuric acid disodium salt
11	810	↑	498.2891	aCDCA, tauroursodeoxycholic acid, tauroursodesoxycholic acid
12	810	↑	514.2843	aTaurocholic acid
13	1050	↑	437.206	aCDCA deoxycholic acid
14	1110	↑	448.3074	aChenodeoxycholic acid glycine conjugated, glycine conjugated deoxycholic acid
15	1290	↓	476.2773	UK
16	1350/1410	↓	540.3302	UK
17	1350	↓	564.3304	UK
18	1470	↓	566.3485	UK
19	1590	↓	568.3623	UK
20	1770	↓	433.236	5,6-Dihydroxy-prostaglandin F1A

CDCA, chenodeoxycholic acid; DCA, deoxycholic acid; GCA, glycocholic acid; PBC, primary biliary cirrhosis; HCs, healthy control subjects.

a Denotes bile acids. Unmarked markers represent carnitines. UK, unknown.
